# Study Protocol: *Transition_psy* a Multicenter Prospective Longitudinal Cohort Study Assessing Risk and Protective Factors to Develop Psychopathology in Transitional Age Youth in Belgium

**DOI:** 10.3389/fpsyt.2021.645679

**Published:** 2021-06-21

**Authors:** Joana Reis, Simone Marchini, Anthony De Leeuw, Hichem Slama, Christophe Leys, Marie Delhaye, Charles Kornreich, Hélène Nicolis, Véronique Delvenne

**Affiliations:** ^1^Faculty of Medicine, Université Libre de Bruxelles, Brussels, Belgium; ^2^Child and Adolescent Psychiatry Department, Queen Fabiola Children's University Hospital, Brussels, Belgium; ^3^Mental Health Service, Université Libre de Bruxelles, Brussels, Belgium; ^4^Child and Adolescent Psychiatry Department, Erasme Hospital, Brussels, Belgium; ^5^Psychiatry Department, Brugmann University Hospital, Brussels, Belgium; ^6^Neuropsychology and Speech Therapy Department, Erasme Hospital, Brussels, Belgium; ^7^Faculty of Psychological Sciences and Education, Université Libre de Bruxelles, Brussels, Belgium

**Keywords:** transitional age youth, child and adolescent psychiatry services, adult psychiatry services, psychopathology, dimensional approach

## Abstract

**Introduction:** Emerging adults are a particularly at-risk population in mental health. The primary aim of the *Transition_psy* study is to evaluate changes in mental health care need and quality of life during transition from Child and Adolescent Mental Health Services (CAMHS) to Adult Mental Health Services (AMHS). The relationship between these changes and genetic or environmental vulnerabilities and clinical dimensions representing risk and protective factors to the development of psychopathology will be analyzed. We also aim to explore how each factor plays, specifically, a role in developing internalizing and externalizing symptoms, in order to predict the most common paths of psychopathology in transitional age youth (TAY).

**Methods and Analysis:** *Transition_psy* is a multicenter prospective longitudinal cohort study. The transversal and trans-diagnostic approach consists of a dimensional evaluation: 300 youth at the age of 17 will be included in a cohort of in-patients, out-patients and control group. Participants will be assessed at baseline (T0) and 24 months later (T1). The primary objective to determine changes in self-rated Health Of The Nation Outcome Scales For Children And Adolescents (HONOSCA-SR) and WHO Quality of Life-BREF (WHOQOL-BREF) scores between T0 and T1. Pearson correlation and mediation analysis will be performed. A secondary objective analysis using mediation and moderation models with several dimensional aspects, including self-reported and cognitive measures, will be conducted to disentangle the potential relationships between the two scores.

**Discussion:** Transition from CAMHS to AMHS occurs at a crucial age in terms of the continuum between adolescent and adulthood psychopathology. This collaborative and cohesive protocol between CAMHS and AMHS represents the first national cohort study about Transition Psychiatry in French-speaking Belgium.

**Ethics and Dissemination:** The study protocol was approved by the Institutional Review Boards (IRB) of the three participating sites. Results will be published in peer-reviewed journals and disseminated at national and international conferences. This trial was registered in ClinicalTrials.gov (Identifier: NCT04333797) on 3 April 2020.

## Introduction

Adolescents and young adults are a particularly at-risk population in mental health. According to the National Comorbidity Survey Replication, involving more than 9,000 people in the United States between 2001 and 2003, the first psychiatric symptoms appeared before the age of 24 for 75% of the patients, and before 14 for 50% (1). The current hypothesis explaining the occurrence of many psychopathologies in adolescents and young adults is multifactorial, including particularities of the adolescent brain maturation processes and psychosocial and/or environmental factors (2). However, life-long psychiatric coverage is the lowest between 16 and 24 (3).

Child and adolescent psychiatric consultations after the age of 16, and even more after the age of 18, decrease significantly (4).

As a result of these epidemiological observations and by analogy with the management of chronic somatic diseases such as diabetes mellitus or cystic fibrosis in pediatrics, the concept of transition between child and adolescent mental health services (CAMHS) and adult mental health services (AMHS) was created. Transition is defined as “the purposeful, planned movement of adolescents and young adults with chronic physical and medical conditions from child-centered to adult-oriented health-care systems” (5).

The TRACK study, conducted in England, on the transition from CAMHS to AMHS showed that only 5% of young people that reach the age of transition to adult services would experience an effective and optimal transition. Different types of difficulties may explain why young people face a barrier when they could benefit from care in an adult psychiatric setting. The threshold age for acceptance in adult services and for cessation of care in child psychiatry varies greatly, from 16 to 21 years. In addition, youth in transition with neurodevelopmental disorders (especially ADHD, learning disabilities and autism spectrum disorders) are less often supported by adult services (6). Young people also feel that the devices are too stigmatizing and they are not familiar with existing care options (7).

However, an interruption of adolescent care for patients with chronic psychiatric conditions is of poor prognosis for their future development and is associated with various social problems: school drop-out, higher unemployment rate, lack of housing, incarcerations, substance abuse, pregnancies or risk behaviors. Early treatment, for example for psychosis, is associated with a better prognostic of the course of the disease (8–10). As there is a fracture in the care system between CAMHS and AMHS, transitional age youth (TAY) fall into a “hole” because of the lack of articulation and adequacy of the health system.

To improve transition from CAMHS to AMHS, the Milestone project, launched in 2014 and ended in 2019, inventories practices in eight European countries to map transitional policies across the European Union (EU) and developed a model of transitional care (11). A randomized controlled trial compared youth in transition with a mental health problem supported by this model of transitional care, and youth in transition with a mental health problem supported by usual care (11).

The categorical approach, usually used in clinical research, is based on a list of signs and symptoms which draws a clear line between normal and psychopathological state, according to an arbitrarily defined threshold (12). This type of approach is increasingly criticized for multiple reasons: excessive comorbidity between the different syndromes, social, cultural and environmental context is not considered, and developmental trajectory is not evaluated. Finally, the diagnosis used, such as depression, post-traumatic stress disorder and so on, would not represent valid disease entities based on any pathophysiological substrate. These observations would explain the gap between neuroscientific discoveries and clinical practice which has remained unchanged for years (13, 14).

Dimensional approaches integrating psychopathology and neuroscience are a new point of view in the field of psychiatry. Developed in 2009 by the National Institute of Mental Health (NIMH), the framework Research Domain Criteria (RDoC) defines psychopathology according to a dimensional approach. RDoC is a matrix of elements: a function or a symptom presents a continuum of variation ranging from its absence to its most extreme expression (12, 15). The matrix contains six major domains: negative valence systems, positive valence systems, cognitive systems, systems for social processes, arousal/regulatory systems and sensorimotor systems. Inside each domain there are “constructs” which correspond to different dimensions, for example working memory, attention, reward responsiveness or acute threat. RDoC takes also into account the development trajectories and the environment as dimensions (15). However, few data exist about clinical dimensional characteristics involved in the development of psychopathology during the transition period and to identify at-risk youth.

*Transition_psy* study is a longitudinal prospective study that aims to evaluate changes in mental health care need and quality of life during transition from CAMHS to AMHS. Since these changes are potentially connected with different vulnerabilities, such as genetic or environmental ones, we will analyse the role of these factors in the emergence of psychopathology in TAY (**Figure 2**). Different dimensions, such as cognitive and executive functions, emotional regulation, social adaptation and quality of care, will be assessed in order to determine whether they are risk or protective factors to develop psychopathology and how they impact quality of life and mental health care need in TAY. We additionally aim to explore how each factor plays, specifically, a role in developing internalizing and externalizing symptoms, in order to predict the most common paths of psychopathology in TAY.

## Methods and Analysis

### Study Design

*Transition_psy* is a multicenter prospective longitudinal cohort study of youth aged 17 years old to assess risk and protective factors to develop psychopathology in TAY.

Assessments will be carried out twice in 2 years [time 0 [T0], at baseline and time 1 [T1], 24 months later] on a clinical and non-clinical sample of youth. The age of 17 years has been chosen to ensure that data collection occurred over the Belgium's CAMHS/AMHS transition boundary of 18 years old. Clinical and non-clinical population will be assessed to establish risk and protective factors to develop psychopathology during transitional age.

### Study Setting

Recruitment settings will be clinical and non-clinical. Thus, participants will be recruited among both help-seeking or diagnosed youth and non-help-seeking participants, that represents control and possibly at-risk participants.

Clinical settings will consist in both outpatient and inpatient facilities within the urban area of Brussels, Belgium. Participants will be recruited from three general hospitals (Queens Fabiola Children's University Hospital [HUDERF], Brugmann University Hospital [CHU Brugmann] and Erasmus Hospital [CUB Erasme]), and one fully outpatients clinic (Mental Health Service at ULB [SSM-ULB]).

Participants from non-clinical settings will be recruited from the general population and from Youth Aid Residential Services (YRS) in the urban area of Brussels.

### Study Population

Eligible participants are either male or female and 17 years old at the time of baseline assessment. All participants must be sufficiently fluent in French. Both parents or legal holder of parental authority and the participant must be able to provide informed and written consent.

The exclusion criteria are applicable to participants diagnosed with a progressive and severe illness that affects short-term vital prognosis (e.g., cancer, cardiac failure, renal failure, central nervous system disorder…). Among help-seeking youth, the mental health professional in charge of the participant's care informs the research assistant (RA) if the subject meets the eligibility criteria. Those who are expected to be unable to answer the assessment tools or with a previously documented homogenous intelligence quotient (IQ) below 75 will be excluded from the study. The active participation in another research study is also an exclusion criterion. All eligibility criteria at the time of baseline assessment are summarized in Table 1.

**Table 1:** Eligibility criteria at T0.

**Inclusion criteria**	**Exclusion criteria**
Male or Female	Diagnosed with a progressive and severe illness that affects short-term vital prognosis
17 years old	Impossibility to answer the assessment tools
French-speaking	Previously documented homogenous IQ < 75 and/or incapacity to answer the assessment tools
Informed and written consent provided by parents or legal holder of parental authority	Active participation in another research study
Informed and written consent provided by the participant	

### Sampling, Recruitment, and Consent

**Figure 1:**
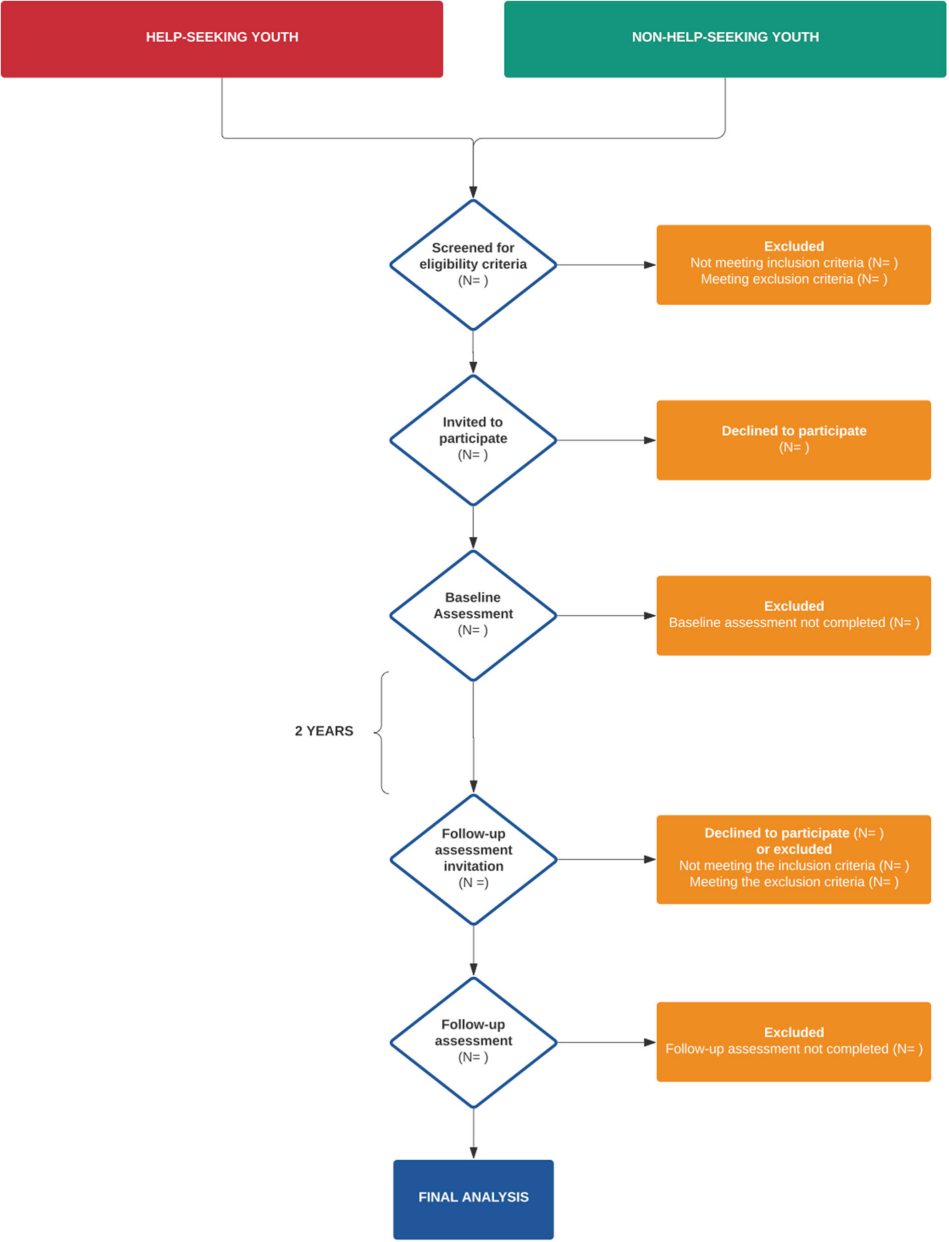
Recruitment plan flowchart.

The recruitment plan (Figure 1) has been structured, with two different recruitment paths for clinical and non-clinical settings, respectively. From the Institutional Review Board (IRB) approval at each site, recruitment of all eligible participants will last 12 months. A total group of 300 youth aged 17 years old, equally subdivided for each study site, will participate. This age will ensure that youth will cross the CAMHS/AMHS transition boundary during the study.

Among help-seeking youth, at T0, the mental health professional from one of the clinical settings will propose to the participants aged 17 years old and their parents or legal holder of parental authority to participate in the study, if they meet the eligibility criteria. If they agree to first contact, a RA will send an invitation letter. Between 7 and 15 days after having sent the letter, one of the investigators will contact by phone call to verify that the subject is effectively eligible to participate.

Non-help-seeking participants, at T0, will be recruited through posters and flyers shortly describing the study. The youth could easily signal their interest to the RA. Once they showed their interest, the RA will phone call them to verify that they are effectively eligible to participate.

Concerning both clinical and non-clinical recruitment paths, if eligibility criteria are fully filled, the RA will organize a meeting with the adolescent and the parents or legal holder of parental authority to provide the study information and answer questions. If the youth prefers to discuss the project at a later time, they will be given the study information and the RA will contact them later on via the preferred method of contact. If the youth agrees to participate, the informed consent is signed. Once the youth agrees to participate and the consent is obtained, the RA sets up a time to conduct the initial interview.

At T1, we foresee contacting all participants and conducting data collection in person, in the same settings as in T0, or remotely, according to participants' preferences.

Recruitment for the study presented here started from the IRB approval on May 2020 at each site. Baseline recruitment is scheduled to conclude in June 2021. It is anticipated the final participant will complete all assessments in June 2023.

### Data Collection

Data collection will take place at T0 and T1. At T0, the participants will complete self-report questionnaires and will be submitted to a cognitive functions assessment. The baseline evaluation, comprised of two different instances, will take ~90–120 min. At T1, the assessment will consist of self-report questionnaires. The follow-up evaluation will take ~30–60 min.

### Measures and Outcomes

Outcome measures were determined based on a literature review of all suitable measures and their psychometric properties. Psychometric properties will be assessed in our sample for each outcome measure through internal coherence parameters (i.e., Cronbach's alpha coefficients). All the outcome measures were previously validated in French.

The primary outcome measures are the self-rated Health Of The Nation Outcome Scales For Children And Adolescents (HONOSCA-SR) (16, 17) and the WHO Quality of Life-BREF (WHOQOL-BREF) (18, 19). Both HONOSCA-SR and WHOQOL-BREF scores will be compared between T0 and T1.

The secondary outcome measures include two distinct types of measures. The first ones consist of self-reported measures evaluating genetic and environmental vulnerability, internalizing and externalizing symptoms, and potential risk and protective factors to develop psychopathology in TAY and/or to increase the participant's level of mental health care need during transition. The second ones assess the cognitive functions of the participants.

All outcome measures at both T0 and T1 are summarized in Table 2.

**Table 2:** Summary information on measures collected at T0 and T1.

**Measure name [Reference]**	**Time point**		**Report on**	**Domain**
	**T0**	**T1**		
**Primary Outcomes**			
Self-rated health of the nation outcome scales for children and adolescents (HONOSCA-SR) (1)	X	X	SR^a^	Care need^c^
World health organization quality of life—bref (WHOQOL-BREF) (2)	X	X	SR^a^	Quality of life^c^
**Secondary Outcomes**			
General questionnaire	X	X	SR^a^	Sociodemographic/Personal and family medical history^c^
Barratt impulsiveness scale version 11 (BIS-11) (20)	X		SR^a^	Symptoms/Behavior^c^
Beck cognitive insight scale (BCIS) (21)	X		SR^a^	Insight^c^
Beck Depression Inventory—Ii (BDI-II) (5)	X		SR^a^	Symptoms^c^
Beck scale of suicide ideation (BSS) (22)	X		SR^a^	Symptoms^c^
Behavior rating inventory of executive function—self report (BRIEF-SR) (23)	X		SR^a^	Executive functions^c^ Cognitive systems—cognitive control^d^
Childhood trauma questionnaire−28 (CTQ-28) (24)	X		SR^a^	Trauma^c^ Negative valence systems—sustained threat^d^
Emotion regulation questionnaire for children and adolescents (ERQ-CA) (25)	X		SR^a^	Emotion regulation^c^
Family assessment device−12 (FAD-12) (9)	X		SR^a^	Family environment^c^
General health questionnaire−12 (GHQ-12) (10)	X		SR^a^	Mental health^c^
Helping alliance questionnaire for child and parents (haq-cp) (20, 25)		X	SR^a^	Therapeutic alliance^c^
Social adaptation self-evaluation scale (SASS) (11)	X	X	SR^a^	Social adaptation^c^
Stroop task (15–17)	X		NC^b^	Executive functions^c^
Test of attentional performance version 2.3.1 (TAP 2.3.1)—subtests alertness, divided attention, flexibility and working memory (26)	X		NC^b^	Executive functions^c^
Tower of london test (TOL) (18, 19)	X		NC^b^	Executive functions^c^
Transition readiness and appropriateness (TRAM) []	X		SR^a^	Transition readiness^c^
Transition related outcomes (TROM) []		X	SR^a^	Transition outcome^c^
Wechsler adult intelligence scale 4th edition (WAIS-IV) (13, 22)	X		NC^b^	IQ^c)^
Youth self report 11–18 (YSR/11–18) (12)	X		SR^a^	Symptoms/Behavior^c^

*See the main text for a description of measures*.

*Report on*:

a *Self-Report Measure*,

b *Neurocognitive Testing; Domain*:

c *General Domain*,

d *Research Domain Criteria (RDoC)*.

#### Primary Outcomes

Participants will complete the HONOSCA-SR (16, 17), a 13-item self-report questionnaire scored on a five- point scale (0–4), which measures the severity of physical, personal and social problems of children and young people with mental health problems. The total score, ranging from 0 to 52, represents the overall severity of care need. To ensure consistency and comparability between T0 and T1 scores, HONOSCA-SR is used throughout, instead of switching to the adult version, the Health Of The Nation Outcome Scales (HONOS).

Quality of life will be assessed by the WHOQOL-BREF (18, 19), a 26-item self-report questionnaire on a five-point scale (1–5). This instrument measures four broad domains—physical health, psychological health, social relationships and environment. Each domain includes several questions that are considered together in the calculation of each domain score. In addition, the WHOQOL-BREF also contains two questions to assess rated quality of life and satisfaction with health. For each domain, higher scores represent higher levels of quality of life.

#### Secondary Outcomes

##### Baseline Assessment (T0)

At T0, some global data, regarding personal and family medical history (including detailed psychiatric history, presence of a psychiatric disorder and/or a psychotropic treatment) and the socio-demographic characteristics of the participants and their families, will be collected in a general database.

The General Health Questionnaire−12 (GHQ-12) (22, 27), a 12-item self-report tool scored on a 4-point scale (0–3), will be used as a screening device for identifying non-psychotic and minor psychiatric disorders. Total scores range from 0 to 36 with higher scores indicating more psychological distress.

Participants will complete the Youth Self-Report 11–18 (YSR/11-18) (20, 25), a 112-item questionnaire on a three-point Likert scale (0–2). This instrument assesses eight empirically derived syndrome scales (anxious/depressed, withdrawn/depressed, somatic complaints, social problems, thought problems, attention problems, rule breaking behavior, aggressive behavior) that can be converted in two higher order factors—global internalizing and externalizing problem behavior score.

Depressive symptoms will be evaluated by the Beck Depression Inventory—II (BDI-II) (24, 28), a 21-item self-report measure on a four-point scale (0–3). Total score ranges from 0 to 63 and higher scores represent more severe depressive symptoms. Suicidal ideation will be examined in detail using the Beck Scale of Suicide Ideation (BSS) (29, 30), a 19-item self-report measure on a three-point scale (0–2 points). Total score ranges from 0 and 38 and higher scores indicate a greater risk of suicide.

Youth will complete the Barratt Impulsiveness Scale Version 11 (BIS-11) (26, 31), a questionnaire designed to assess the personality and behavioral construct of impulsiveness. This 30-item self-report measure is composed of 30 items, which are scored on a four-point scale (1–4). Higher total scores represent higher levels of impulsivity.

The Emotion Regulation Questionnaire for Children and Adolescents (ERQ-CA) (32) will be used to evaluate two emotion regulation strategies: emotion regulation by cognitive reappraisal (CR) and/or expressive suppression (ES). ERQ-CA is a 10-item self-report questionnaire (where six items relate to CR whereas the remaining four relate to ES) scored on a five-point Likert scale (1–5). Total scores for each subscale range from 6 to 30 and from 4 to 20, for CR and ES, respectively. For each of the scales, higher scores indicate a greater use of the corresponding strategy.

Participants will complete the Childhood Trauma Questionnaire−28 (CTQ-28) (33, 34), a 28-item self-report questionnaire scored on a five-point Likert scale (1–5), which measures trauma during childhood. CTQ is divided in five subscales (emotional, physical and sexual abuse, and emotional and physical neglect) with a total score ranging from 28 to 140. Higher scores represent higher intensity of childhood trauma.

Family functioning will be evaluated by the Family Assessment Device−12 (FAD-12) (23, 35), a 12-item self-report questionnaire on a four-point scale (1–4), with a total score ranging from 12 to 48. Higher scores represent worse levels of family functioning. Additionally, social adaptation and interpersonal relationships will be assessed by the Social Adaptation Self-evaluation Scale (SASS) (36, 37), a 21-item self-report questionnaire on a four-point scale (0–3). Total scores range from 0 to 60 with higher scores representing higher social adaptation.

At T0, a cognitive functions assessment (including intellectual and executive functions) will be conducted, using the Wechsler Adult Intelligence Scale 4th edition (WAIS-IV) (21), the Test of Attentional Performance version 2.3.1 (TAP 2.3.1) (38), the Stroop Task (39–42), the Tower of London Test (TOL) (43, 44) and the Behavior Rating Inventory of Executive Function—Self-report (BRIEF-SR) (45, 46). An estimate of the participant's intelligence quotient (IQ) will be calculated based on a validated short form of the WAIS-4 (47), including four subtests: vocabulary, similarities, figure weight and matrix reasoning. Participants presenting an IQ estimation below 75 will be excluded from the statistical analysis. Four TAP 2.3.1 subtests will be used to assess alertness, divided attention, flexibility, and working memory. Cognitive inhibition will be assessed by the Stroop task and planning task will be assessed by the TOL. The BRIEF-SR, a 75-item self-report questionnaire, assesses the adolescent's view of his or her cognitive, emotional and behavioral functions. BRIEF-SR consists of two major index subscales and a composite of the two, which are the Behavioral Regulation Index (BRI), the Metacognition Index (MI), and the Global Executive Composite (GEC). BRI comprises inhibit, shift and emotional control scales, while MI comprises initiate, working memory, plan/organize, organization of materials and monitor scales. Higher scores are positively related with lower levels of executive functions. Cognitive insight will be measured by the Beck Cognitive Insight Scale (BCIS) (48, 49), a 15-item self-report questionnaire on a four-point scale (0–3). This instrument contains two sets of items: a nine-item self-reflectiveness subscale and a six-item self-certainty subscale. A composite index is calculated by subtracting the score for the self-certainty scale from the score for the self-reflectiveness scale.

##### Follow-Up Assessment (T1)

At T1, some global data, regarding participant's social inclusion, autonomy and psychiatry follow-up between T0 and T1, will be collected in a general database. Social adaptation and interpersonal relationship will be reassessed, using the SASS (36, 37).

The perception of therapeutic alliance will be tested by the child version of the Helping Alliance Questionnaire for Child and Parents (HAQ-CP) (50, 51), a 15-item self-report questionnaire on a six-point scale (1–6), including two sets of items: a 13-item therapeutic alliance subscale and a two-item overall psychological state subscale. The overall score for the therapeutic alliance ranges from 13 to 78 with higher scores representing higher levels of therapeutic alliance.

### Descriptive Statistics

With regard to categorical sociodemographic variables, summary tabulations of absolute and relative frequencies in each category of qualitative variables will be presented. Missing data will be treated, in the first instance, with pairwise deletion. After data collection, in case that missing completely at random data are believed to be predominant, we will proceed to listwise deletion. If missing at random data are more plausible, regression imputation will be used.

With regard to continuous sociodemographic variables, the number of participants, mean, median, standard deviation, minimum and maximum values, and interquartile ranges will be presented. Mean and standard deviation will be used as central tendency and dispersion indicators when the distribution is approximately normal, with medians and interquartile ranges used otherwise. The normality of the distributions will be assessed with graphical displays (histogram, box plot and normal plot).

### Data Analysis and Sample Size

**Figure 2:**
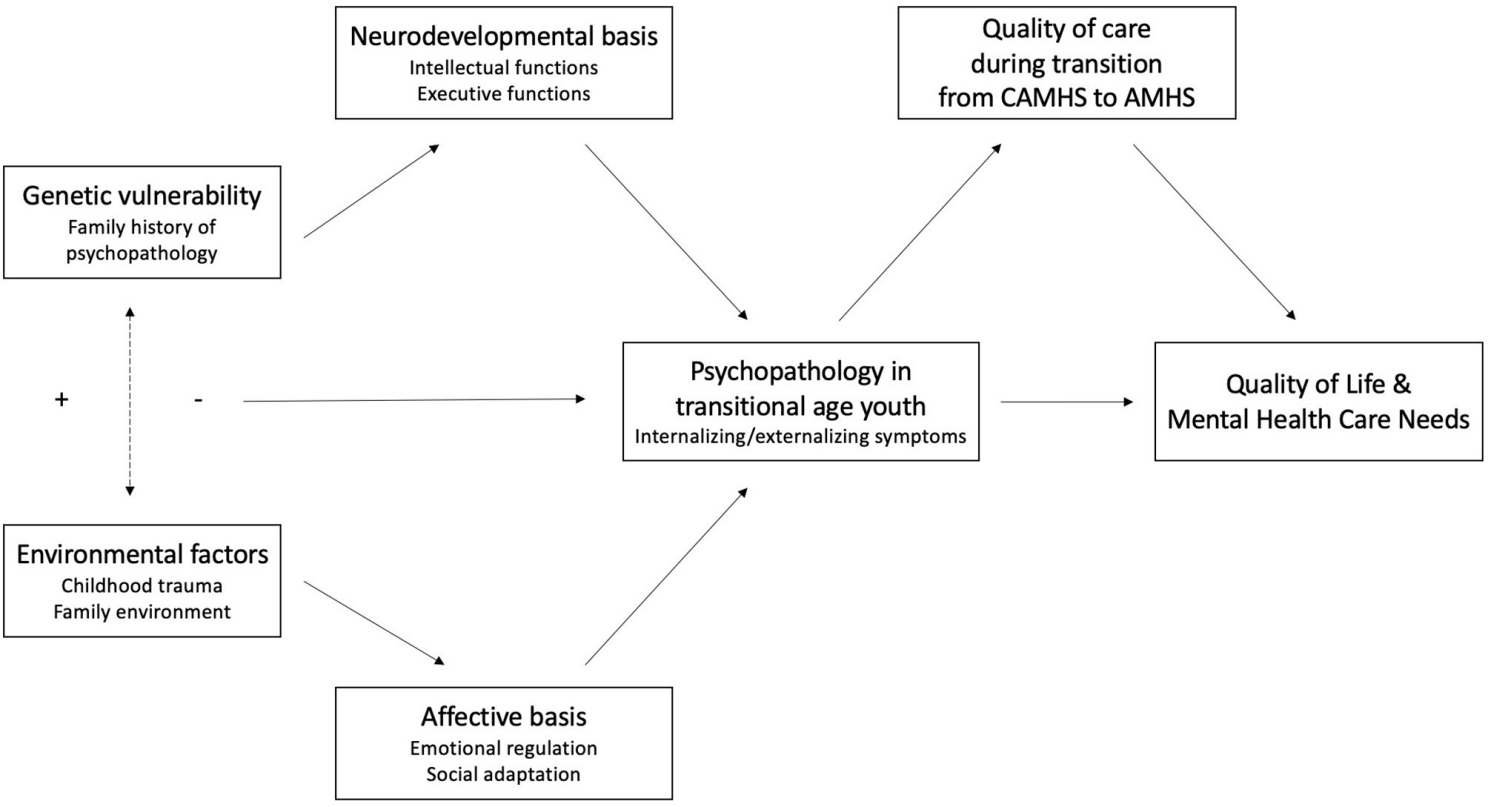
Conceptual model of the primary aim.

Regarding our conceptual model, earlier illustrated in Figure 2, genetic vulnerability and environmental factors (independent variables), will be, respectively, assessed by self-reported psychiatric family history and both CTQ-28 and FAD-12 questionnaires. Firstly, we expect that youth's psychopathology during the transition period, assessed by YSR/11–18 internalizing and externalizing subscales, GHQ-12, BDI-II, BSS and BIS-11, will play a role as a mediator between previously described independent variables and change in quality of life and mental health care need between T0 and T1 (dependent variables). Thus, these variables over the transition period, evaluated by HONOSCA-SR and WHOQOL-BREF, will allow to define distinct trajectories. Secondly, dimensional aspects will be analyzed as moderating factors, representing protective or risk factors in this process. The evaluated dimensional aspects are intellectual and executive functions (WAIS-IV, TAP 2.3.1, Stroop Task, TOL, BRIEF-SR, BCIS), emotional regulation (ERQ-CA), social adaptation (SASS) and quality of care (HAQ-CP).

Regarding our primary objective, two kind of analysis will be conducted: Pearson correlation and mediation. We computed an *a priori* power analysis using G^*^Power software to estimate the sample size for these tests. To reach a medium effect size (i.e., r = 0.3), with an α-error of 5% (two-sided) and a β-error of 80%, the total sample size represents 84 participants for Pearson correlations and 149 participants for mediation analysis (based on a three groups IV and one mediator). We decided to recruit 300 youth in our study to allow for up to half of the participants dropping out of the study.

To reach our secondary objective, we will first identify the most powerful predictors between risk and protective factors with linear regression analysis. Then, to assess the weight of each risk and protective factor identified with linear regression in the relation between the difference of HONOSCA-SR scores and WHOQOL-BREF scores between T0 and T1, mediation/moderation analyses will be conducted using the PROCESS macro in SPSS (52).

### Methodological Considerations

#### Attrition Bias

Loss to follow-up is inherent in longitudinal studies. High retentions rates (>70%) with similar populations have been demonstrated in recent follow-up management models (53).

Our retention rate plan includes a detailed contact management plan and age-appropriate communication with youth. To ensure that contact with the participant is not lost during the T1 phase and that data are as complete as possible, the research team will exhaustively collect contact details, including postal addresses, telephone numbers and e-mail addresses of participants and parents or legal holder of parental authority. At T1, we foresee contacting participants and conducting data collection in person or remotely, according to participants' preferences.

#### Bias in Recruitment

At T0, participants are asked to attend sessions with the RA on two separate occasions for ~1 h for each visit, representing a great time commitment. Thus, many participants who are interested in participating in the study could be unable to attend the study. It is possible that we are recruiting a biased sample with only participants who can easily move to the RA. To address this issue, current efforts are directed toward expanding the hours of the study and enhancing the online component where participants may complete questionnaires at home.

#### Assessment and Inter-rater Training

Regarding RA's training and performance, we followed a three-phase process (54). Firstly, each RA has been trained by senior trainers, within our team, to use all the tools. Secondly, senior trainers evaluated the performance of each RA. Thirdly, the research team organized inter-rating sessions to ensure neuropsychological assessments' reliability. Regarding this last step, for every 50 participants, all RA will assign scores to the same assessment, with the supervision of a senior trainer. Cohen's kappa will be calculated to assess inter-rater reliability.

### Data Management and Confidentiality

Data on study participants will be entered in an electronic case report form (CRF) in a secured database located on the leading site (Research Electronic Data Capture—REDCap). In REDCap database, participants are coded, and study data entered into the CRF is only accessible by authorized persons. Data will be stored for 25 years.

### Patient and Public Involvement

Patients and general public were not involved in the design, recruitment or conduct of the study. All study participants were informed that questionnaire results will not be disclosed to them, to their parents/legal holder of parental authority or to their healthcare providers before the end of T1 phase.

Study participants were informed that after completion of the study and publication of the data, they are free to contact the research team to be informed about questionnaire results.

We provide to each participant and their legal holder of parental authority, a document listing all possible and accessible resources in mental health, such as anonymous and free helplines for mental health support and one specifically for suicide prevention; free access and low-cost mental health services for adolescents and young adults in our region; psychiatric emergency departments; outpatients psychiatric facilities contacts and all team members contacts. In the event that the completion of questionnaires or the cognitive functions assessment causes a psychological distress, signaled by the participant or their legal holder of parental authority, all RA are trained to accompany them to contact a mental health care professional or even to contact directly one, if the participant prefers so.

## Discussion

Transition from CAMHS to AMHS occurs at a crucial age in terms of the continuum between adolescent and adulthood psychopathology. However, there is an evident lack of coverage and articulation between CAMHS and AMHS and literature lacks sufficiently powerful studies and randomized controlled trials to build protocols on the transition. There is also limited knowledge on the dimensional characteristics behind the development of mental health disorders during this period.

*Transition_Psy* study is a large collaborative project between CAMHS and AMHS with a dimensional research approach from controls to in and out-patients in the transition period. This study is the first prospective longitudinal cohort study about transition psychiatry in French-speaking Belgium.

The main goal of this study is to provide evidence about changes in self- rated mental health care need and quality of life over the transition period and its associations with both genetic or environmental vulnerabilities and emergence of psychopathology in TAY. This research has the potential to contribute to identify clinical dimensional characteristics as risk or protective factors to the development of psychopathology, as well as targets for early intervention and prevention efforts. The knowledge gained from this study could lead to the implementation of improvements in clinical practice. The final purpose is to develop a non-stigmatizing approach to reduce rejection from youth in transition in psychopathological suffering and increase social inclusion.

## Ethics Statement

The studies involving human participants were reviewed and approved by Queens Fabiola Children's University Hospital, Brussels, Belgium; Brugmann University Hospital, Brussels, Belgium; Erasme Hospital, Brussels, Belgium. Written informed consent to participate in this study was provided by the participants' legal guardian/next of kin.

## Author Contributions

VD was the chief investigator who conceptualized the study and wrote the grant funding proposition, together with MD, HN, CK, and JR. JR, SM, and ADL prepared the first draft and subsequent versions of the protocol and this manuscript. JR, SM, and ADL are the research assistants and wrote the ethical approvals documents. HS was the neuropsychologist responsible for the selection of neuropsychological assessment tools. CL was the statistician who led the calculation of the sample size and the design of statistical analysis model. All authors reviewed the proposed study methods and reviewed the protocol. VD reviewed all study information material before submission to ethics and this manuscript. All authors approved the submitted version.

## Conflict of Interest

The authors declare that the research was conducted in the absence of any commercial or financial relationships that could be construed as a potential conflict of interest.
